# A Comparative Study on Subcutaneous Negative Suction Drain vs. No Drain in Emergency Laparotomy Wounds for Peritonitis

**DOI:** 10.7759/cureus.84247

**Published:** 2025-05-16

**Authors:** Bhaskar Mallaiah, Bhavana Chinmayee K, Ramachandra J

**Affiliations:** 1 General Surgery, Kempegowda Institute of Medical Sciences, Bengaluru, IND; 2 Surgery, Kempegowda Institute of Medical Sciences, Bengaluru, IND

**Keywords:** emergency laparotomy, peritonitis, subcutaneous drains, subcutaneous negative pressure closure, surgical site infection

## Abstract

Aim

This study aimed to compare the effectiveness of subcutaneous negative suction drainage vs. no drainage in reducing postoperative complications following emergency laparotomy for peritonitis.

Objective

The primary objective of this study was to evaluate the effect of subcutaneous negative pressure closure on various postoperative outcomes in patients undergoing emergency surgery. Specifically, the study aims to assess its impact on the incidence of surgical site infection (SSI), wound dehiscence, the need for secondary suturing, and the duration of hospital stay. Additionally, the study seeks to determine whether the observed SSIs are primarily attributable to intra-abdominal infections or are a result of hospital-acquired cross infections.

Materials and methods

A prospective comparative study was conducted on 60 patients diagnosed with peritonitis who underwent emergency laparotomy. They were randomly assigned into two groups of 30 patients each: Group A (with subcutaneous negative suction drain) and Group B (without drain). In Group A, drain fluid was analyzed for infection characteristics, while wound discharge from both groups was assessed microbiologically, where present.

Results

Group A included 21 males (70%) and nine females (30%), while Group B had 26 males (86%) and four females (13%). Midline vertical incisions were used in 90% of cases, with the remaining 10% receiving right paramedian incisions. Discharge from the incision site was observed in five patients (16%) in Group A and 13 patients (43%) in Group B (p = 0.024). SSIs occurred in five patients (16.67%) in Group A and in eight patients (26%) in Group B. Seroma formation was observed in five patients (16.67%) in Group B, but none in Group A. No significant differences were noted in wound dehiscence, need for secondary suturing, or duration of hospital stay between the groups.

Conclusion

Subcutaneous negative suction drainage significantly reduced the incidence of SSI, seroma formation, and discharge from the incision site following emergency laparotomy for peritonitis. This may lower the risk of wound complications and postoperative morbidity. Further randomized controlled trials are recommended to validate these findings.

## Introduction

Surgical site infections (SSIs) and delayed wound healing are frequently observed following abdominal surgeries for secondary peritonitis of varied etiologies, such as perforated appendicitis, gastroduodenal ulcers, or enteric perforations. The severity of wound complications may differ based on the underlying cause, degree of contamination, and host factors [1]. The development of an SSI significantly impacts patient morbidity, mortality, hospital stay, healthcare costs, and patient satisfaction [2,3].

SSIs remain one of the most common healthcare-associated infections, accounting for approximately 20% of all HAIs. Recent estimates suggest that 2% to 5% of patients undergoing inpatient surgery develop an SSI despite advances in infection prevention strategies [1,4]. While preoperative antibiotic prophylaxis and thorough intraoperative peritoneal lavage are well-established measures to reduce infection risk, the technique of wound closure also plays a critical role.

While seroma formation is more frequently associated with fatty or dependent anatomical areas, it can still occur in midline laparotomies, particularly in emergency cases involving peritonitis and subcutaneous dissection. It may lead to further wound complications such as abscess formation, calcification, and poor cosmetic outcomes, including unsatisfactory scar appearance. In cases of SSI, a burst abdomen poses a major clinical concern. Re-closure may impair respiratory function, while leaving the wound open increases the risk of nosocomial infections [5].

Several studies have demonstrated that using negative suction in the subcutaneous plane reduces infection by effectively evacuating contaminated fluid and seromas [6-8]. Negative pressure also promotes wound healing by lowering the bacterial load, providing a moist and protected environment, reducing peripheral oedema, enhancing circulation, and accelerating granulation tissue formation and epithelialization [9,10].

Given this background, the present study was undertaken to compare the effectiveness of subcutaneous negative suction drainage versus conventional wound closure in emergency laparotomy for peritonitis, focusing on the frequency of SSI, wound dehiscence, the need for secondary suturing, and the duration of hospital stay.

## Materials and methods

Study design and setting

This was a randomized, prospective, comparative study conducted in the Department of General Surgery at Kempegowda Institute of Medical Sciences (KIMS), Bengaluru. The study period spanned from November 2018 to October 2020. Prior to commencement, the study protocol received approval from the Institutional Ethical Committee of KIMS.

Study population

The study included adult patients aged 18 years and above who were diagnosed with peritonitis and underwent emergency exploratory laparotomy at KIMS Hospital. Written informed consent was obtained from all participants prior to inclusion in the study.

Inclusion and exclusion criteria

All patients above the age of 18 years who were admitted with clinically and radiologically confirmed secondary peritonitis, due to causes such as gastrointestinal perforation, appendicitis, or enteric fever, and were scheduled for emergency exploratory laparotomy at KIMS Hospital were recruited into the study after obtaining informed consent. Patients were excluded if they were below 18 years of age, were immunocompromised (including those with HIV, uncontrolled diabetes, those on long-term steroid therapy, or those undergoing chemotherapy), were pregnant, or required an ileostomy or colostomy during the surgical procedure. Patients requiring intraoperative formation of stomas (ileostomy or colostomy) were excluded from the study.

Grouping and sample size

A total of 60 patients (calculated using MedCalc® Statistical Software, MedCalc Software Ltd., Ostend, Belgium) who met the eligibility criteria were randomly assigned into two groups(simple randomization technique, sealed envelope method), with 30 patients in each group. Group A comprised patients whose abdominal closure involved placement of a subcutaneous negative suction drain using a Romo Vac No. 16 device (Romsons Group Private Limited, Agra, India). Group B included patients who underwent abdominal closure without the use of a subcutaneous drain. The sample size was determined based on a previous study by Kagita et al. [10], which reported SSI rates of 12.5% in the intervention group and 69.4% in the control group. With a type I error of 1%, a power of 95%, and a 10% buffer to account for non-responsiveness, the final calculated sample size was 30 patients per group.

Preoperative and operative management

All patients received standard preoperative care, including resuscitation, nasogastric decompression, correction of fluid and electrolyte imbalances, and empirical administration of broad-spectrum antibiotics consisting of a third-generation cephalosporin, an aminoglycoside, and metronidazole. The diagnosis of peritonitis was established based on clinical features, laboratory investigations, and imaging studies such as abdominal and chest X-rays to detect signs like free intraperitoneal air, bowel dilatation, or paralytic ileus, which were interpreted in conjunction with clinical findings. The majority of patients underwent surgery within six to 12 hours of admission. Emergency exploratory laparotomy was performed under strict aseptic precautions by experienced surgeons from the same unit of the department. After appropriate intraoperative management and peritoneal lavage using normal saline, closure of the rectus sheath was accomplished with non-absorbable Loop PDS 1-0 sutures. In Group A, a single Romo Vac No. 16 (Romsons Group Private Limited, Agra, India) subcutaneous drain was inserted in the lower subcutaneous plane and brought out through a separate stab incision lateral to the main surgical wound. No intraperitoneal drains were used in either group. The skin was then closed using either Ethilon 2-0 mattress sutures or staples. In Group B, the abdominal closure was performed in a standard fashion without insertion of a subcutaneous drain.

Postoperative assessment

Postoperative evaluation focused on identifying SSIs, wound dehiscence, need for secondary suturing, duration of hospital stay, and occurrence of seroma formation. SSIs were diagnosed according to the Centers for Disease Control and Prevention (CDC) criteria [11]. In Group A, a subcutaneous Romo Vac drain was used, and daily output was recorded. Samples from the subcutaneous drain fluid were sent for culture and sensitivity testing on postoperative day 2 or day 3 and at the time of drain removal (when output was <25 mL for two consecutive days). In Group B, if any wound discharge was observed, it was similarly cultured to assess superficial infection. These assessments focused solely on subcutaneous contamination and were not intended to represent intra-abdominal pathology. To determine the source of infection, culture results of intra-abdominal fluid and wound discharge were compared on postoperative day 5 or day 6. Seromas were managed through drainage and antiseptic dressings, whereas wound dehiscence was managed conservatively with secondary suturing after sufficient granulation tissue formation.

Statistical analysis

All data were analyzed using SPSS (IBM SPSS Statistics for Windows, IBM Corp., Version 22, Armonk, NY). Categorical variables were summarized as frequencies and proportions, and comparisons were made using the chi-square test. Continuous variables were expressed as mean values with standard deviations and analyzed using the independent t-test. A p-value of less than 0.05 was considered statistically significant.

## Results

A total of 60 patients diagnosed with peritonitis and undergoing emergency exploratory laparotomy at Kempegowda Institute of Medical Sciences were enrolled in this study. These patients were randomly assigned to two equal groups of 30 each.

Demographic characteristics

Gender Distribution

The gender distribution showed that out of the total 60 patients, 47 were male, and 13 were female. In Group A, 21 patients (70%) were male, and nine (30%) were female, while Group B comprised 26 males (86.7%) and four females (13.3%). Although males predominated in both groups, the difference in gender distribution was not statistically significant (p = 0.117; Table 1).

**Table 1:** Gender distribution between the two groups Comparison of sex distribution between Group A and Group B was done using the chi-square test. χ² - chi-square value; df - degrees of freedom

Sex	Group A	Group B	Total	χ²	df	p-value
Male	21 (70%)	26 (86.7%)	47 (78.3%)	2.455	1	0.117
Female	9 (30%)	4 (13.3%)	13 (21.7%)	-	-	-

Age Distribution

Age distribution across the two groups revealed that in Group A, the majority of patients were in the 51-60 years age group (23.3%), while in Group B, the 21-30 years age group was most common (23.3%). There was no statistically significant difference in age distribution between the two groups (p = 0.410; Table 2).

**Table 2:** Age distribution between the two groups Age group distribution was compared using the chi-square test. χ² - chi-square value; df - degrees of freedom

Age Group (Years)	Group A	Group B	Total	χ²	df	p-value
≤20	1 (3.3%)	2 (6.7%)	3 (5.0%)	-	-	-
21-30	4 (13.3%)	7 (23.3%)	11 (18.3%)	6.119	6	0.41
31-40	4 (13.3%)	1 (3.3%)	5 (8.3%)	-	-	-
41-50	5 (16.7%)	6 (20.0%)	11 (18.3%)	-	-	-
51-60	7 (23.3%)	6 (20.0%)	13 (21.7%)	-	-	-
61-70	3 (10.0%)	6 (20.0%)	9 (15.0%)	-	-	-
>70	6 (20.0%)	2 (6.7%)	8 (13.3%)	-	-	-

Comparing the Mean Age

When comparing the mean age, Group A had a mean age of 51.43 ± 16.80 years, and Group B had a mean age of 48.13 ± 17.77 years. The difference was not statistically significant (t = 0.738, p = 0.463; Table 3).

**Table 3:** Mean age comparison Mean age was compared using the independent t-test. SD - standard deviation; t - t-value

Group	Mean Age (Years)	SD	t-value	p-value
Group A	51.43	16.8	0.738	0.463
Group B	48.13	17.77	-	-
Total	49.78	17.23	-	-

Comparison of the incision types used between the two groups

The type of incision used during surgery was predominantly midline in both groups. In Group A, 28 patients (93.3%) underwent midline laparotomy, and two (6.7%) had a right paramedian incision. In Group B, 26 patients (86.7%) had a midline incision, and four (13.3%) underwent a right paramedian incision. There was no significant difference in incision type between the groups (p = 0.367; Table 4).

**Table 4:** Type of incision used between groups Incision type distribution between groups was compared using the chi-square test. χ² - chi-square value Note: No stomas were performed in either group. Each Group A patient received one subcutaneous Romo Vac drain placed in the lower part of the incision, exteriorized through a separate stab incision.

Incision Type	Group A	Group B	Total	χ²	p-value
Midline	28 (93.3%)	26 (86.7%)	54 (90.0%)	0.814	0.367
Right paramedian	2 (6.7%)	4 (13.3%)	6 (10.0%)	-	-

Incidence of wound discharge

The incidence of wound discharge was significantly lower in Group A. Only five patients (16.7%) in Group A had discharge from the incision site, compared to 13 patients (43.3%) in Group B. This difference was statistically significant (χ² = 5.079, p = 0.024; Table 5).

**Table 5:** Incidence of wound discharge between groups Comparison of wound discharge incidence between the groups was done using the chi-square test. χ² - chi-square value

Wound Discharge	Group A	Group B	Total	χ²	p-value
No	25 (83.3%)	17 (56.7%)	42 (70.0%)	-	-
Yes	5 (16.7%)	13 (43.3%)	18 (30.0%)	5.079	0.024*

Comparison of the culture growth between the intra-operative peritoneal fluid and post-operative wound discharge/drain fluid

Among patients with wound discharge, culture comparison was done between intraoperative peritoneal fluid and postoperative wound discharge or drain fluid. In Group A, one patient (3.3%) showed growth identical to intra-abdominal infection, and four patients (13.3%) had hospital-acquired organisms. In Group B, three patients (10.0%) had similar growths to intraoperative specimens, five (16.7%) had hospital-acquired infections, and five (16.7%) showed no growth. The difference in culture patterns between the groups was not statistically significant (p = 0.506; Table 6).

**Table 6:** Culture growth analysis in patients with wound discharge Comparison of culture patterns between intra-abdominal and wound discharge samples was performed using the chi-square test. χ² - chi-square value

Culture Type	Group A	Group B	χ²	p-value
Same as peritoneal fluid culture	1 (3.3%)	3 (10.0%)	-	-
Hospital-acquired (cross-infection)	4 (13.3%)	5 (16.7%)	-	-
No growth	0 (0%)	5 (16.7%)	0.442	0.506

Incidence of wound dehiscence and need for secondary suturing between the two groups

The incidence of wound dehiscence was low and similar across both groups. In Group A, one patient (3.3%) developed wound dehiscence requiring secondary suturing, while in Group B, two patients (6.7%) had wound dehiscence. This difference was not statistically significant (χ² = 0.351, p = 0.554; Table 7).

**Table 7:** Incidence of wound dehiscence and need for secondary suturing Wound dehiscence rates were compared using the chi-square test. χ² - chi-square value

Dehiscence	Group A	Group B	χ²	p-value
No	29 (96.7%)	28 (93.3%)	-	-
Yes	1 (3.3%)	2 (6.7%)	0.351	0.554

Mean duration of subcutaneous suction drain placement in group

Among patients in Group A, the average duration of subcutaneous suction drain placement was 6.13 ± 1.04 days (Table 8).

**Table 8:** Mean duration of subcutaneous suction drain (Group A only) Descriptive statistics presented for drain duration. No inferential test applied.

Group	Mean Duration (Days)	SD
Group A	6.13	1.04

Duration of hospital stay

The mean duration of hospital stay was 10.80 ± 3.27 days in Group A and 11.77 ± 6.12 days in Group B. This difference was not statistically significant (t = 0.762, p = 0.449; Table 9).

**Table 9:** Duration of hospital stay Comparison of hospital stay duration between the groups was performed using the independent t-test. SD - standard deviation; t - t-value

Group	Mean (Days)	SD	t-value	p-value
Group A	10.8	3.27	-	-
Group B	11.77	6.12	-	-
Total	11.28	4.89	0.762	0.449

SSI and seroma formation in both groups

Regarding postoperative complications, five patients (16.7%) in Group A developed SSI, and none had seroma formation. In contrast, Group B had eight patients (26.7%) with SSI and five patients (16.7%) with seroma formation. Both SSI and seroma incidence were significantly higher in Group B (χ² = 5.301 and 4.462, respectively, p = 0.0218 for seroma and p = 0.0347 for SSI (Table 10).

**Table 10:** Incidence of SSI and seroma formation Comparison of complication rates was done using the chi-square test. χ² - chi-square value; SSI - surgical site infection

Complication	Group A	Group B	χ²	p-value
Seroma	0 (0%)	5 (16.7%)	5.301	0.0218
SSI	5 (16.7%)	8 (26.7%)	4.462	0.0347

## Discussion

Our results indicate that the use of subcutaneous drainage significantly reduces wound-related complications such as discharge, SSIs, and seroma formation, although it showed no statistically significant difference in terms of wound dehiscence and hospital stay.

A major finding of this study was the significantly lower incidence of wound discharge in the subcutaneous drain group (Group A, 16.67%) compared to the conventional closure group (Group B, 43.33%) (p = 0.024). This is consistent with studies such as those by Gupta and Kumar [12] and Owens and Stoessel [6], which reported reduced wound complications with the use of negative pressure wound therapy. Vashist et al. also noted improved wound outcomes when negative suction was used for abdominal wall closure in sepsis [13].

When comparing the etiology of wound infections, we found that hospital-acquired cross-infections were more prevalent than infections originating from the peritoneal cavity. Only 3% of infections in Group A and 10% in Group B were linked to intra-abdominal sources, while 13% and 16%, respectively, were due to nosocomial transmission. This finding reinforces the importance of rigorous postoperative infection control practices, especially in emergency surgical settings.

Regarding wound dehiscence and need for secondary suturing, although Group A had a lower rate (3.33%) than Group B (6.67%), the difference was not statistically significant. Chowdri et al. reported similar observations in obese patients, advocating the use of subcutaneous drains to reduce dehiscence and infection risk [14].

One of the most noteworthy results in our study was the complete absence of seroma formation in Group A, compared to 16.7% in Group B. This supports the evidence from Harish et al., who documented significantly reduced seroma rates in patients managed with subcutaneous suction drainage [15]. Sumi et al. also reported reduced seroma and SSI rates in patients with colorectal perforation who received subcutaneous closed suction drains [16].

The incidence of SSI was lower in Group A (16.67%) than in Group B (26.67%). Although not statistically significant, this trend aligns with the findings of Gupta and Kumar [12], who showed a protective effect of suction drains in abdominal surgeries. Similar benefits of negative pressure closure in reducing SSIs have been reported by Stannard et al. [7] and Willy et al. [9], who emphasized its clinical value in high-risk surgical wounds and contaminated fields.

As for hospital stay, our study found a mean duration of 10.8 days in Group A and 11.7 days in Group B, which was not statistically significant. This contrasts with the findings of Naik et al. [17], who observed earlier discharge and faster recovery in patients receiving subcutaneous negative pressure closure. However, hospital stay is multifactorial and can be significantly influenced by the severity of the primary pathology, systemic complications, and comorbidities, which may explain the lack of significant difference observed between the two groups.

In conclusion, our findings support the use of subcutaneous negative suction drains in emergency laparotomy wounds. They significantly reduce wound discharge, seroma formation, and SSIs. Although they may not significantly affect hospital stay or dehiscence rates, the overall trend favors improved wound outcomes with drains. These results are consistent with multiple prior studies and advocate for the routine use of drains in contaminated surgical wounds.

This study has several limitations. Firstly, the sample size was relatively small, with only 60 patients enrolled, which may limit the generalizability of the findings. Secondly, the study was conducted at a single tertiary care center, which may introduce institutional bias. Thirdly, microbiological differentiation between intra-abdominal and hospital-acquired infections was based solely on culture reports without molecular typing, which may affect diagnostic accuracy. Additionally, due to the emergency nature of surgeries, intraoperative contamination and surgical technique were not fully standardized. Finally, the short duration of follow-up confined to the inpatient period may have missed delayed SSIs or complications.

## Conclusions

Subcutaneous negative suction drainage facilitates improved wound healing by actively reducing bacterial load and eliminating dead space. In our study, patients who underwent emergency laparotomy for peritonitis and received subcutaneous negative suction drains demonstrated a significant reduction in the incidence of SSIs, seroma formation, and wound discharge.

By minimizing postoperative wound complications, this intervention has the potential to reduce overall patient morbidity. However, to substantiate these findings further and establish definitive clinical guidelines, larger multicentric randomized controlled trials are recommended.
